# Recognition of Bookmark Aging Degree Based on Probabilistic Neural Network

**DOI:** 10.1155/2022/3151441

**Published:** 2022-02-16

**Authors:** Cong Zheng, Xiaoling Zhang, Shaoqiu Ma, Zhijian Xiao

**Affiliations:** The School of Mathematical Engineering, Zhejiang Dongfang Polytechnic, Wenzhou 325000, China

## Abstract

Bookmarks are the basis for librarians to get books on and off shelves and borrowers to borrow books. In order to solve the problem of time-consuming and labor-consuming manual checking of bookmark aging, this paper proposes a method of bookmark aging recognition based on image processing technology. First, we perform image preprocessing, Otsu threshold segmentation, and morphological processing on the acquired bookmark image to obtain the effective area of the bookmark, then acquire the aging features for the bookmark, and finally input the acquired features into the trained neural network for defect recognition. The experimental results show that the method proposed in this paper can achieve 96% recognition, which can more accurately identify the aging defects of bookmarks.

## 1. Introduction

With the proposal and continuous innovation of the concept of smart library [1], the trend of intelligent digitalization of libraries has become increasingly obvious. Different from digital library [2], smart library emphasizes the intelligent management of library. Besides the management of digital resources, it is necessary to develop an intelligent system for paper book management, so as to enhance the state detection and ensure paper books are in good condition. At present, automatic borrowing and returning of books has been realized, that is, readers can not only check the borrowing status and information of the required books through the mobile phone app or the computer provided by the library but also complete the borrowing and returning procedures in the self-service book borrowing and returning machine [3].

Libraries often check books through inventory, so that the problems of books, such as misplaced books [4], damaged books, and bookmark falloff, can be found [5]. For example, the school library of the author is closed for arrangement every Friday morning. For libraries with large collections, this method will not only affect the normal borrowing of books but also pose great challenges to librarians. In 2016, more than 100 librarians in Nanjing University Library spent 5 days to complete the inventory of above 900,000 books [6]. In view of the shortcomings of traditional library management, such as being time-consuming and labor-consuming, having low efficiency, and common complaints of “unable to find books,” some libraries have developed library robots, such as the intelligent book inventory robot in Nanjing University Library, the RFID book automatic inventory robot in Wuhan University Library [7], and the intelligent chat robot Xiaotu in Tsinghua University Library [8]. Library robot has the advantages of saving manpower, noncontact, time management, and efficiency, which can solve the problems of traditional library management to a certain extent. Particularly, in terms of manpower, time, and cost, it can fundamentally improve the library management and make routine management possible. However, according to the current studies and reports, some library robots can only realize the handling in designated positions, such as the handling robots of Humboldt University Library in Germany and Osaka City University Library in Japan [9]. Other library robots need to customize bookshelves or use other devices to keep books in the designated state, such as the automatic access robots of Johns Hopkins University in the United States [10] and National University of Singapore [11]. The workload and cost of bookshelf update and modification are massive. Although the current robot technology has been relatively mature, it can hardly be popularized in library management. Therefore, for the current large libraries, it is more appropriate to apply robot technology to library management based on the current actual situation of libraries than to completely adopt new technology. According to the existing studies of intelligent book management, the combination of robots and other technologies is more suitable. In this study, the book state detection algorithm is mainly developed based on image processing technology, which can be superimposed on the robot. The robot is equipped with a camera to realize the image collection. The proposed algorithm is used to detect and judge book images and provide corresponding suggestions to assist the daily management and maintenance. Different book detection algorithms will be continuously developed for later robot implantation, so as to effectively reduce the times of internal arrangement by closing library and decrease the workload of librarians in library management.

## 2. Book Detection Framework

**Figure 1:**
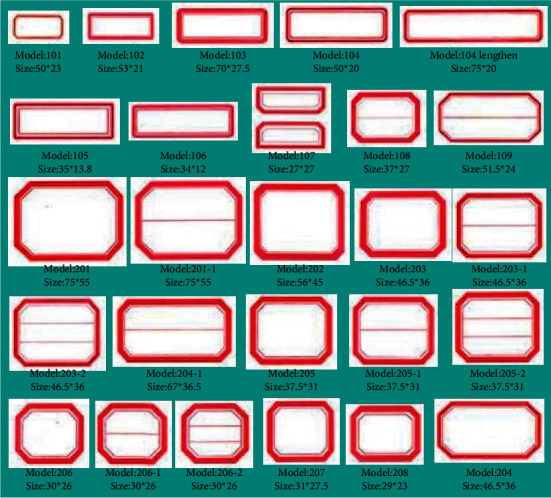
Bookmark types.

As a bridge among books, readers, and librarians, bookmarks play a vital role in book borrowing and book shelving.  Figure 1 shows several common types of the bookmark.

**Figure 2:**
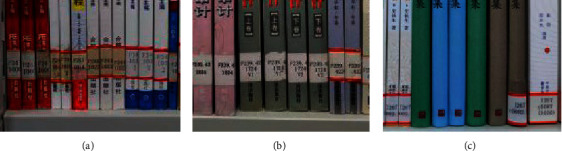
The common problems of bookmarks. (a) The problem of aging. (b) The problem of damage. (c) The problem of falloff.

Different from the two-dimensional code of books, the bookmark is usually attached to the lower part of the spine. It is easily exposed to the air and affected by the frequent circulation of books, resulting in aging, damage, and even falling off. Some common problems of bookmarks are shown in Figure 2.

**Figure 3:**
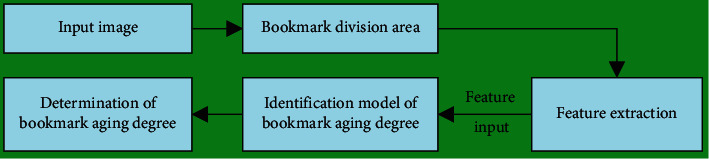
The overall flow of the bookmark detection algorithm.

A aging bookmark detection algorithm based on image processing is proposed, whose overall flow is shown in Figure 3.

Firstly, image segmentation is carried out for the obtained bookmark image, the bookmark area is segmented, and five features of aging classification are extracted from the segmented bookmark area, which are gray mean, median and variance of B channel, and the mean and median of S channel. The extracted features are input to the trained neural network model, and three aging states of bookmarks are obtained, namely, non-aging, slight aging, and severe aging.

## 3. Detection of Bookmark Aging

According to the bookmark images, bookmarks usually have the following characteristics:

- Bookmarks are composed of the white background with black characters, and there are edges of other colors with a certain width.
- When bookmarks are aged, they will become yellow or even brown. The more serious the aging, the darker the color.

**Figure 4:**
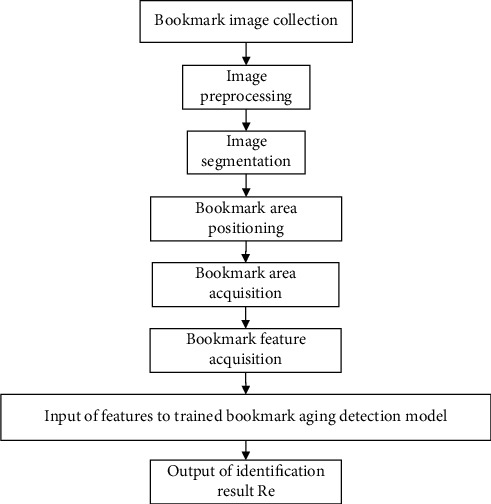
The bookmark aging detection algorithm.

Based on the above-mentioned characteristics of bookmarks, a bookmark aging detection method based on visible light images is proposed. With the robot as the carrier, a high-definition pan/tilt camera is used to shoot bookmark images. First, the bookmark area is extracted by image preprocessing, segmentation, and extraction algorithm. Second, according to the color characteristics, the aging characteristics of bookmarks are determined. Finally, the aging degree of bookmarks is judged by inputting the bookmark characteristics to the trained aging detection model. The flowchart is shown in Figure 4.

### 3.1. Bookmark Segmentation

**Figure 5:**
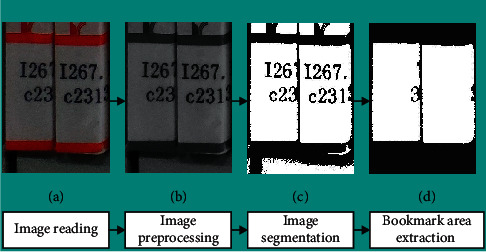
The process of the bookmark area extraction.

Before aging detection, it is necessary to extract the label from the image, and the integrity of the label region extraction is the premise to ensure the aging recognition accuracy. Therefore, before detecting the aging of bookmarks, this study first determines the bookmark area. The main methods include image preprocessing, image segmentation, and morphological processing.  Figure 5 shows the process of label region extraction.

- *Image Preprocessing*. Preprocessing of bookmark images [12] is not only the premise of bookmark aging but also an important step of bookmark recognition and aging degree detection. The main purpose is to make bookmarks obvious in images, so as to separate bookmarks and remove the background information. Firstly, the images are grayed and converted from RGB images to gray images, which is conductive to simplifying subsequent detection process and decreasing computation burden.
- *Image Segmentation and Morphological Processing*. After preprocessing the collected bookmark images, the bookmark area is preliminarily extracted by image segmentation [13], and the segmented images are subjected to morphological processing [14] to remove the bookmark's noisy point. Meanwhile, bookmarks are separated from the background according to the unique distribution characteristics, thus determining the bookmark area.

Because of the special structural features of bookmarks, a unified brightness map is obtained through camera imaging, and the light reflection ability is stronger than that of the background. After image preprocessing and image segmentation, an accurate bookmark area can be obtained, which provides a guarantee for subsequent processing.

### 3.2. Aging Degree Detection

**Figure 6:**
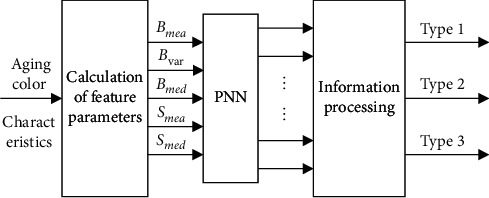
The model of the bookmark aging detection.

Aging is inevitable in the use process, but the causes are complex. Aging problem cannot be solved fundamentally. Therefore, the best solution is to regularly check and then replace aged bookmarks. In this study, neural network is used to classify the aging degree. Before detection, relevant characteristic parameters are extracted, and bookmarks are detected by using PNN (probabilistic neural network). The detection mode is shown in Figure 6.

#### 3.2.1. Feature Extraction

Because of obvious color changes caused by bookmark aging, the recognition of the aging degree can be transformed into color recognition. The gray values of R, G, and B channels [15] will decrease during aging, and the change of B channel is the most obvious. HSI color space [16] is composed of hue (H), saturation (S), and intensity (I) separately. S component is less affected by external factors, and its performance is relatively stable. Therefore, this study selects B and S components to extract feature values [17] (including mean value, median value, and variance) of the two channels (B and S) in the bookmark area, respectively (five features, namely, *B*_*mea*__,_*B*_var__,_*B*_*med*__,_*S*_*mea*_, and *S*_*med*_). The feature calculation formula is as follows.

Mean value:
  (1) $$\begin{array}{c} G_{mea}=\sum_{i=1}^{m}\sum_{j=1}^{n}g_{i,j}p_{i,j}. \end{array}$$
Median value:
  (2) $$\begin{array}{c} G_{med}=\underset{1\leq i\leq m,1\leq j\leq n}{\text{median}}g_{i,i}. \end{array}$$
Variance:
  (3) $$\begin{array}{c} G_{\mathrm{var}}=\sum_{i}{\left(g_{i,j}-G_{mea}\right)}^{2}p_{i,j}, \end{array}$$
where *g*_*i*,*j*_ represents the gray values in the B and S images; *p*_*i*,*j*_ is the probability of gray value *g*_*i*,*j*_; *m* and *n* represent the image size; and *g*_*i*,*j*_ is the operation of taking the median value.

According to the field test, three groups of sample data of bookmark aging degree can be obtained, as shown in Table 1. The second column of Table 2 refers to the three aged bookmarks obtained from the collected image (the aging degree is non-aging, slight aging, and severe aging), and the corresponding five feature values are listed in the third to eighth columns, respectively. It can be found that the extracted features have obvious differences in different aging degrees, and it is effective to select the five features for classification.

#### 3.2.2. Aging Detection Model

**Figure 7:**
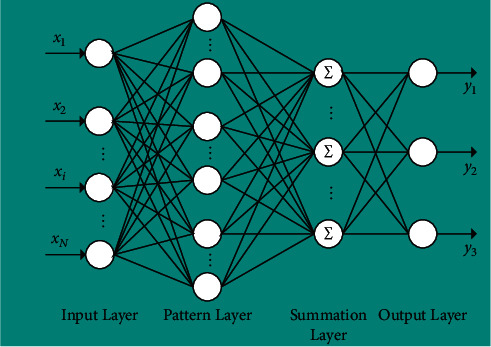
Probabilistic neural network structure diagram.

PNN (probabilistic neural network) [18] is a feed-forward artificial neural network put forward by Dr. Specht in 1990 on the basis of Bayes rule and radial basis neural network. It is composed of radial basis neuron [19] and competition neurons [20]. As shown in Figure 7, the model is mainly composed of four layers: input layer, pattern layer (training set), summation layer, and output layer. It can use linear learning algorithm to complete the function of non-linear algorithm while maintaining the high precision of non-linear algorithm. With the characteristics of simple structure and quick training, the model is suitable for pattern classification.

*(1) Classification of Probabilistic Neural Networks*.

Input layer: the input layer receives data and transmits them to the mode layer. The number of input neurons is the same as the dimension of input vector.
Pattern layer: the pattern layer calculates the matching between input vector and each pattern of the training set. The number of training samples determines the number of neurons in the pattern layer. Neurons correspond to training samples. Each pattern layer calculates the output result according to the following formula:
  (4) $$\begin{array}{c} \phi_{ij}\left(x\right)=\frac{1}{{\left(2\pi \right)}^{d/2}\sigma^{d}}\left[-\frac{{\left(x-x_{ij}\right)}^{T}\left(x-x_{ij}\right)}{2\sigma^{2}}\right], \end{array}$$
where *σ* is the smoothing coefficient; *x* is the input vector composed of calculated color features; and *x*_*ij*_ is the neuron vector of the *i*_th_ pattern layer of class *j*.
Summation layer: neurons in the summation layer correspond to the classification patterns, which are connected with the pattern layer through sparse links. The connection condition of neurons between the summation layer and the pattern layer is that they correspond to the same classification pattern. By summing the output values, the maximum possibility of a certain category can be obtained. The calculation formula is shown in the following formula:
  (5) $$\begin{array}{c} p_{j}\left(x\right)=\frac{1}{{\left(2\pi \right)}^{d/2}\sigma^{d}}\frac{1}{N_{j}}\sum_{i=1}^{N_{j}}\exp\left[-\frac{{\left(x-x_{ij}\right)}^{T}\left(x-x_{ij}\right)}{2\sigma^{2}}\right], \end{array}$$
where *N*_*j*_ is the number of samples of class *j*.
Output layer: the class with the highest posterior probability is selected as the classification result according to Bayesian decision rules, and the calculation formula is as follows:
  (6) $$\begin{array}{c} C\left(x\right)=\arg\max\left\{p_{j}\left(x\right)\right\},\;j=1,2,\ldots ,m, \end{array}$$
where *C*(*x*) is the estimation class of the input vector *x* and *m* is the total number of classes.

*(2) Construction and Training of Network*. There are five nodes in the input layer of PNN (probabilistic neural network), which correspond to five characteristic parameters (*B*_*mea*_, *B*_var_, *B*_*med*_, *S*_*mea*_, and *S*_*med*_). There are three nodes in the output layer, which correspond to three states, namely, non-aging, slight aging, and severe aging. These three states are expressed in binary format, as shown in Table 2.

100, 010, and 001 represent the categories of non-aging, slight aging, and severe aging bookmarks output by the network, respectively. In the subsequent testing process, in order to draw the test result diagram, the description corresponding to the aging category will be converted into decimal representation, that is, 1, 2, and 3 represent 100, 010, and 001, respectively.

## 4. Algorithm Experiment

In order to verify the effectiveness of the proposed bookmark aging detection method based on the PNN (probabilistic neural network), experiments were carried out on a large number of collected bookmark images, including image segmentation, neural network training and testing, and aging degree detection. The experiment was completed on a computer with Intel Core i5-3240 @3.40 GHz CPU and 4G memory and image processing software MATLAB R2014a.

### 4.1. Bookmark Segmentation Effect

The bookmark segmentation proposed in this paper is mainly based on maximum between-class variance (Otus method) threshold segmentation and morphological filtering. The segmented bookmark area is mapped back to the original image to obtain the bookmark area without background interference. The processing results are shown in Table 3. The second to sixth lines represent the original image, the grayed image, the threshold segmented image, the morphologically processed image, the bookmark area determined by the minimum circumscribed rectangle, and the color image of the bookmark area.

According to the Otsu segmentation results in Table 3, it can be found that the bookmarks aged in different degrees are correctly segmented, and the whole area is complete without false segmentation. The proposed segmentation method is effective and feasible for the bookmark segmentation.

### 4.2. Aging Degree Identification Test

**Figure 8:**
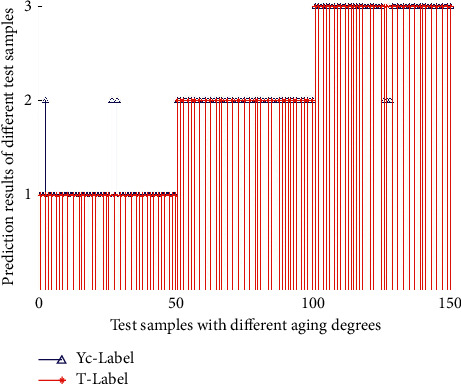
Comparison of PNN's detection and actual situation.

In this study, the bookmark images taken in the experimental environment are selected. 60 images of non-aging, slight aging, and severe aging samples are taken as the training samples, respectively. The input layer of the neural network is (*B*_*mea*_, *B*_var_, *B*_*med*_, *S*_*mea*_, *S*_*med*_), and the output layer has three aging degrees (nonaging, slight aging, and severe aging). *B*_*mea*_, *B*_var_, *B*_*med*_, *S*_*mea*_, and *S*_*med*_ of the training sample are extracted as the input of the neural network, and the aging degree is the output for PNN neural network training. 150 images of samples with different aging degrees (50 images of non-aging, slight aging, and severe aging in each category) are selected as the test samples to simulate the neural network, and the corresponding aging degree discrimination results are output. There are obvious differences among bookmarks with different aging degrees in terms of *B*_*mea*_, *B*_var_, *B*_*med*_, *S*_*mea*_, and *S*_*med*_. The actual output and the target output of 150 test samples are shown in Figure 8, in which Yc-Label is the experimental test result and T-Label is the actual test result.

As can be seen from Figure 8, all slight aging bookmarks are correctly identified, and three non-aging bookmarks and severe aging bookmarks are mistakenly identified as slight aging. In pairwise transition, the values of the extracted features may be similar, and the typical samples fail to cover adequately, resulting in recognition errors. Such problems can be solved by accumulating the number of samples.

In this study, the backpropagation (BP) neural network and the competition network are also simulated and tested. As shown in Table 4, the accuracy of the neural network is 96%, which is obviously higher than that of the BP neural network [21] and the competition network, thus realizing the accurate judgment of the gray density degree of bookmark aging.

This study selects five bookmark images to analyze the gray aging degree of bookmarks, which are compared with the field manual test results. The results are shown in Table 5. The proposed method can detect and judge the actual aging degree of bookmarks, and the results have good consistency.

## 5. Conclusions

The bookmark defect detection method based on probabilistic neural network can make full use of the prior knowledge of faults and qualitatively detect the aging of bookmarks under Bayesian minimum risk criterion. Probabilistic neural network has high training speed. It is easy to implement in engineering and has strong robustness and high recognition accuracy. With the gradual accumulation of fault knowledge, the network can be continuously expanded to further improve the detection accuracy.

This study proposed the identification of bookmark aging degree based on probabilistic neural network and applied it to detect the aging degree of bookmarks. The identification accuracy is 96%, which is higher than that of the traditional method. The proposed method lays a foundation for the detection of bookmark defects by using image processing technology.

However, the proposed method only analyzes the aging of bookmarks, but there are many other defects in bookmarks. Therefore, the algorithm will be improved to further study other types of bookmark defects.

## Figures and Tables

**Table 1:** The data of the aging bookmark.

Serial number	Original image	Type	*B*_*mea*_	*B*_var_	*B*_*med*_	*S*_*mea*_	*S*_*med*_
1	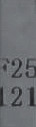	Non-aging	181.22	8.13	188	0.0372	0.0416
2	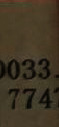	Slight aging	107.96	6.78	115	0.2453	0.2447
3	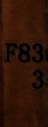	Severe aging	46.17	2.94	47	0.6077	0.6226

**Table 2:** The classification of aging modes.

Aging classification	Description of binary format	Description of decimal format
Non-aging	100	1
Slight aging	010	2
Severe aging	001	3

**Table 3:** The process of the image segmentation.

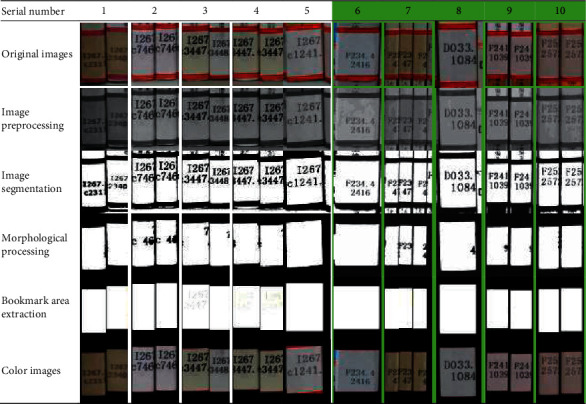

**Table 4:** The result of aging degree judgment.

Aging degree	Actual number	Correctly judged number		
		Competition network	BP	PNN
Non-aging	50	50	44	47
Slight aging	50	37	45	50
Severe aging	50	41	45	47
Total	150	128	134	144
Recognition rate	—	85.33%	89.33%	96%

**Table 5:** The process of image recognition.

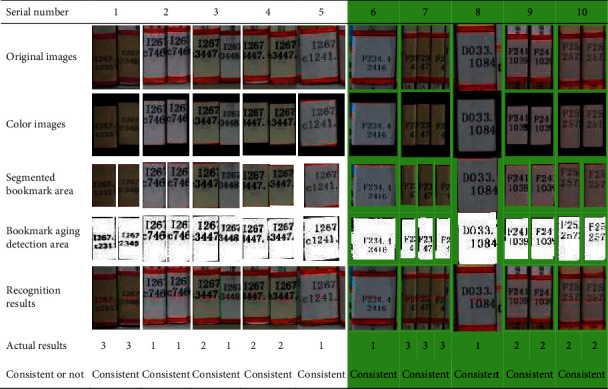

## Data Availability

The characterization data used to support the results and discussion are available from the corresponding author upon request.
